# University of Maryland, Dental Department

**Published:** 1895-06

**Authors:** 


UNIVERSITY OF MARYLAND, DENTAL DEPARTMENT.—The Annual Commencement of 1895 was held at the Academy of Music on Friday afternoon, March 29th. The mandamus was read by the Dean, Prof. Ferdinand J. S. Gorgas.

The degree of "Doctor of Dental Surgery" was conferred upon the following members of the graduating class by the Hon. John P. Poe, Attorney General of the State of Maryland, owing to the illness of the Provost, Bernard Carter, Esq.:

Walter C. Arthur, Pennsylvania; William B. Ashbrook, Pennsylvania; A. Clagett Baker, West Virginia; Louis O. Bartlett, Cuba, W. I.; N. Grant Bowbeer, Canada; Luther D. Blackwelder, Pennsylvania; Albert L. Bumgarner, Pennsylvania; William J. Carter; Georgia; William W. Cavers, New York; J. Calvin Criswell, Pennsylvania; Benjamin F. Copp, Colorado; J. Marten Fleming, Jr., North Carolina; John S. Geiser, Pennsylvania; Timothy O. Heatwole, Virginia; Harvey C. Henderson, North Carolina; David Edward Hoag, New York; John S. Hegner, Switzerland; John Haughton Ihrie, North Carolina; Samuel J. Irvine, Texas; George Franklin Jernigan, Texas; Benedict Kritchevsky, Russia; William H. Kuich, Pennsylvania; Charles William Link, West Virginia; George H. Lyndon, Ohio; Arthur N. McKeever, West Virginia; George C. Mann, Virginia; Charles F. Matthews, California; Edgar H. Markley, Pennsylvania; Raymond W. Palmer, Virginia; Fred. L. Parker, South Carolina; D. Harry R. Patton, Nebraska; John A. Rawlinson, Brazil, S. A.; Stanley C. Reynolds, Canada; B. Franklin H. Rouse, Maryland; Louis St. Clair Saunders, Nova Scotia; George E. Starr, Maryland; J. Albert Stults, New Jersey; Edward A. Tigner, Georgia: George S. Tigner, Georgia; N. McKendree Wilson, Maryland; Cyrus A. Ward, Ohio; George N. Ward, New York; Maynard E. Williams, New York; Sheldon H. Wolf, Pennsylvania.

The address to the graduates was delivered by the Rev. Dr. C. Herbert Richardson, and the Class Oration by D.

Edward Hoag, D. D. S., of New York. The Prizes were awarded by Prof. Francis T. Miles, M. D.

University Gold Medal for highest number of votes, Timothy O. Heatwole; Honorable Mention, Geo. F. Jernigan; S. S. White Prize, Sheldon H. Wolf; Snowden & Cowman Prize, J. Sidney Couret; Dr. J. H. Marchant's Prize, Geo. F. Jernigan; Prof. Harris' Prize, L. St. Clair Saunders; Prof. Gorgas' Prize, Cyrus A. Ward; Dr. John C. Uhler's Prize, D. Edward McConnell; Dr. Isaac H. Davis' Prize, N. Grant Bowbeer; Dr. Clarence J. Grieves's Prize, Geo. F. Jernigan; S. S. White 2nd Prize, Marion N. King; E. C. Moore's Prize, John P. Best.

Students grading over 600 out of a possible 700: T. O. Heatwole, G. F. Jernigan, D. E. Hoag, G. C. Mann, W. H, Kuich, W. C, Arthur, E. A. Tigner, J. M. Fleming, Jr., W. W. Cavers, G. S. Tigner.

The whole number of matriculates for the session was 186.

